# Music-based emotional regulation and nutrition strategies for mental health support among medical student-athletes

**DOI:** 10.3389/fnut.2026.1937687

**Published:** 2026-09-23

**Authors:** Jie Tang, Xianhui Xu

**Affiliations:** College of Music, Xiangnan University, Chenzhou, Hunan, China

**Keywords:** digital phenotyping, emotional balance, food-photo coding, medical student-athletes, music regulation, nutrition rhythm

## Abstract

**Background:**

Medical student-athletes face combined academic, clinical, and competitive training demands that may disrupt sleep, dietary routines, emotional regulation, recovery, and cognitive functioning.

**Objective:**

To examine associations between naturally occurring music-based emotional regulation, self-selected nutrition routines, psychophysiological recovery, and mental health-related markers using a non-interventional multimodal digital phenotyping framework.

**Methods:**

Over 8 weeks, 120 medical student-athletes were monitored without prescribed music, diet, therapy, or behavioral intervention. Data included wearable-derived nocturnal heart-rate variability, resting heart rate, sleep–wake rhythm, activity and training load, participant-recorded music use, meal timing, hydration routines, food-photo logs, and brief emotional reflections. Three indices were constructed: The Music Regulation Pattern Index, Nutrition Rhythm Stability Index, and Psychophysiological Recovery Balance Index.

**Results:**

Monitoring feasibility was high and index reliability was acceptable. Music-regulation patterns were more strongly associated with emotional balance and focus-related language, whereas nutrition-rhythm stability was more closely associated with sleep regularity, heart-rate variability stability, and recovery balance.

**Conclusion:**

Naturally occurring music-use and nutrition-rhythm behaviors showed distinct associations with emotional and physiological recovery markers. Multimodal digital phenotyping may help identify potentially protective lifestyle patterns in medical student-athletes while preserving an observational, non-causal interpretation and avoiding unsupported treatment claims.

## 1 Introduction

Medical student-athletes are positioned at the intersection of rigorous medical education and competitive sport participation. Medical training requires sustained cognitive effort, long study hours, clinical exposure, frequent examinations, and emotional adaptation to patient-centered learning environments (1). Athletic participation simultaneously requires regular training, physical recovery, competition preparation, discipline, and performance consistency. The combination of academic, clinical, and athletic obligations may create a pressure environment in which emotional regulation, sleep quality, nutrition rhythm, recovery capacity, and mental wellbeing become closely interconnected. Mental health in student-athletes has become an important concern in sports science, education, and health promotion (2). Competitive athletes may experience stress, fatigue, performance anxiety, mood fluctuation, burnout, and impaired recovery when training demands are combined with academic pressure and inadequate lifestyle regulation.

Medical students may experience additional strain from intensive coursework, clinical responsibilities, irregular schedules, and limited recovery time (3). When these demands coexist with structured sport participation, medical student-athletes become a distinctive population requiring targeted research attention. Music engagement is an everyday behavior that may contribute to emotional regulation (4). Students and athletes often use self-selected music to manage stress, increase motivation, support focus, prepare for exercise, recover after training, or regulate mood before sleep. In sport settings, music may influence arousal, attention, perceived effort, motivation, and emotional readiness; in academic settings, it may support concentration or emotional decompression (5). However, studies of music use often rely on retrospective self-report or controlled experimental exposure and may not fully capture natural music-use timing across academic, clinical, and athletic contexts (6). Nutrition is another lifestyle factor linked to recovery and psychological balance. Student-athletes require regular nutrition and hydration to support training adaptation, energy availability, immune function, cognitive performance, and post-exercise recovery (7). Medical student-athletes may experience delayed meals, inconsistent hydration, skipped recovery intake, or late-night eating because of clinical rotations, examinations, laboratory work, and training schedules. For this population, nutrition rhythm, including meal timing, hydration consistency, pre-training intake, post-training intake, and daily regularity, may be as relevant as total dietary composition (8).

Although music and nutrition have both been examined in relation to wellbeing and performance, they are rarely studied together as naturally occurring lifestyle regulation patterns. Many previous approaches rely on retrospective self-report, or intervention designs that assign participants to specific music or dietary protocols (9). The present study was not designed to prescribe music sessions, provide a diet plan, or test a treatment effect. Instead, it observes existing daily behaviors and examines associations among natural music-use behavior, self-selected nutrition routines, wearable-derived recovery indicators, and ecological emotional markers (10).

Digital phenotyping refers to the repeated, individual-level quantification of behavioral, physiological, and contextual characteristics in naturalistic settings using data generated through personal digital devices (11). Unlike conventional assessments obtained at a small number of predefined study visits, digital phenotyping is intended to capture temporally dynamic patterns as they occur in everyday life. In the present study, the term is used in this methodological sense because multiple longitudinal data streams including wearable-derived physiology and sleep, participant-recorded music and nutrition behavior, food photographs, training and academic-context information, and text- or voice-based emotional reflections, were repeatedly collected during participants’ usual academic and athletic routines and integrated at the individual level (12).

The methodological novelty of this study lies in integrating music-based emotional regulation, nutrition rhythm, and psychophysiological recovery within a single non-interventional digital phenotyping framework (13). To make the framework reproducible, the revised manuscript provides explicit mathematical definitions and validation metrics for three indices: the Music Regulation Pattern Index (MRPI), the Nutrition Rhythm Stability Index (NRSI), and the Psychophysiological Recovery Balance Index (PRBI) (14). This approach offers a practical model for identifying protective lifestyle rhythms among medical student-athletes while avoiding clinical treatment claims or prospective intervention assignment.

## 2 Methods

### 2.1 Study design

This study used a non-interventional, naturalistic observational design to examine associations among naturally occurring music-based emotional regulation, self-selected nutrition routines, wearable-derived recovery indicators, and mental health-related markers in medical student-athletes. Participants were not assigned to any music program, dietary plan, psychological therapy, training modification, supplement protocol, or behavioral treatment. The study therefore examined behavioral patterns as they occurred during normal academic, clinical, and athletic routines. The study used a multimodal digital phenotyping framework. For the purposes of this study, digital phenotyping was operationally defined as repeated individual-level characterization of physiological, behavioral, and contextual patterns in participants’ natural environments using data obtained through personal digital devices and digitally recorded participant-generated observations. This terminology was selected because data were collected longitudinally during ordinary academic, clinical, athletic, eating, sleeping, and music-listening routines rather than during isolated laboratory assessments.

The framework incorporated complementary forms of digital measurement. Device-derived data included wearable measures of nocturnal heart-rate variability, resting heart rate, sleep–wake patterns, activity, and recovery-related indicators. Participant-generated digital observations included structured music-use records, meal and hydration timing, food-photo records, training-load entries, academic-context information, and brief text- or voice-based emotional reflections. These streams differed in their degree of participant involvement; therefore, the present framework should not be interpreted as exclusively passive digital sensing. Rather, its combined device-derived physiological monitoring with active participant-generated digital records to construct a temporally resolved representation of everyday behavioral and recovery patterns.

The designation “multimodal” refers to the integration of physiological, behavioral, nutritional, contextual, and emotional data modalities within the same longitudinal framework. Data streams were subsequently synchronized at the participant-day level, allowing within-person changes in lifestyle rhythm, psychophysiological recovery, and emotional wellbeing markers to be examined across the eight-week observation period. Thus, digital phenotyping was used here as an observational measurement framework and not as a diagnostic procedure, clinical intervention, or automated mental-health classification system.

### 2.2 Study setting and participants

Participants were enrolled from medical universities, university-affiliated sport teams, student athletic associations, and clinical education programs. For the purposes of this study, medical student-athletes were defined as students currently enrolled in a medical degree program who participated in organized sport training or competition at least three times per week. The planned sample was 120 participants. Eligibility criteria included age 18 years or older, current enrollment in a medical degree program, participation in structured sport training or competition, willingness to wear a non-invasive device during the monitoring period, willingness to record usual music use and nutrition routines, and ability to provide written informed consent. Exclusion criteria were participation in another structured nutrition, psychological, music-based, or behavioral intervention study; inability to complete digital monitoring procedures; incomplete consent; or medical restrictions preventing usual sport participation (15).

### 2.3 Ethical approval and participant consent

The study protocol was reviewed and approved by the Research Ethics Committee of Xiangnan University before data collection (Approval Letter No. XNU-REC-2025-0186; meeting date: December 27, 2025). The committee reviewed the research plan, informed consent form, questionnaire, participant information sheet, data confidentiality statement, and survey/scale instruments. Written informed consent was obtained from all participants prior to participation. Participants were informed that the study was observational, non-invasive, and minimal risk, and that it did not provide medical treatment, psychological therapy, dietary prescription, or clinical diagnosis. They were also informed of the study purpose, procedures, confidentiality protections, anonymity of responses, data storage arrangements, and their right to withdraw at any stage without academic, clinical, athletic, or personal consequences. All data were anonymized by assigning a unique study code, and identifiable information was stored separately from research data.

### 2.4 Observation period and procedure

Data were collected over 8 consecutive weeks during a regular academic and athletic training period. Before monitoring began, participants attended an orientation session in which wearable use, food-photo recording, music-use logging, training-load recording, emotional reflection entry, privacy protection, and withdrawal procedures were explained. No behavioral advice, nutrition plan, music prescription, or psychological training was provided. Participants continued their usual academic schedules, clinical duties, training programs, eating habits, sleep routines, and music-listening behaviors throughout the observation period.

### 2.5 Wearable device specification and physiological signal acquisition

To address reproducibility, the revised protocol specifies the wearable device and signal window. Physiological monitoring was conducted using the WHOOP Strap 4.0 (WHOOP Inc., Boston, MA, USA). The device captured physiological metrics using wrist-worn photoplethysmography and a three-axis accelerometer for heart-rate, heart-rate variability, sleep–wake estimation, daily strain/activity, and recovery-related outputs. All devices were synchronized before study onset, and device model, firmware/app version where available, smartphone operating system, and synchronization status were recorded for each participant (16). Heart-rate variability was operationalized as the root mean square of successive differences between normal heartbeats (RMSSD), reported in milliseconds. Resting heart rate was reported in beats per minute.

To minimize transient daytime autonomic noise, both RMSSD and resting heart rate were derived from the participant’s overnight sleep period, with the wearable output weighted toward stable deep-sleep/slow-wave sleep periods when available. Sleep duration, sleep timing, sleep regularity, daily activity, strain/training load indicators, and estimated recovery trends were exported at the daily level. Days were considered invalid for physiological analysis if the main sleep episode was missing, wear time was insufficient, or the device signal-quality flag indicated unreliable overnight HRV estimation. Consumer wearables may not fully replace laboratory polysomnography or electrocardiography; therefore, all wearable-derived values were interpreted as ecological indicators rather than diagnostic clinical measurements.

### 2.6 Training load and academic context

Training load was quantified using the session rating of perceived exertion method. Within 30 min after each training session, participants recorded session duration in minutes and perceived exertion on a modified CR-10 scale (12). Session training load was calculated as duration × RPE, consistent with standard sport-science practice. Daily academic context variables included examination period, long clinical day, major deadline, laboratory session, and competition exposure. These contextual variables were included to reduce confounding from changes in academic or athletic demand.

### 2.7 Naturalistic music-use data collection

Music-based emotional regulation was assessed through participants’ naturally occurring, self-selected music-listening behavior using structured participant-completed music-use logs. No music was prescribed or assigned by the investigators, and participants were not instructed to listen to a particular artist, genre, tempo, playlist, duration, or type of music. The recorded data therefore represented participants’ habitual music choices during their normal academic, athletic, recovery, commuting, and leisure activities.

Each listening episode was recorded separately. For every episode, participants documented the approximate time of listening, duration of the listening episode, listening context, and self-labeled purpose of music use. Recording episodes individually allowed music exposure to be characterized not only by whether music was used on a given day but also by listening frequency, episode duration, temporal distribution, and the relationship of listening to relevant daily activities. Multiple listening episodes occurring on the same day were retained as separate records so that patterns such as pre-study, pre-training, post-training, commuting, and pre-sleep music use could be distinguished.

Genre information was recorded as a descriptive characteristic of the participant’s self-selected music. Genre was not treated as an experimental condition and no genre was assigned by the research team. Where available, participants also recorded non-content musical characteristics including genre category, tempo category, and perceived rhythm intensity. These characteristics were used to describe the type of music selected under different behavioral and regulatory circumstances rather than to establish controlled exposure to particular musical styles. Because copyrighted audio files were neither collected nor stored, the analysis focused on participants’ reported listening characteristics and behavioral context rather than direct acoustic analysis of individual tracks.

Listening duration was recorded at the episode level and considered together with listening frequency to characterize the extent of music engagement. Duration was therefore distinguished from frequency: frequency represented how often a participant reported listening episodes, whereas duration represented the length of individual listening episodes. Temporal variables further captured when music was used during the day and whether listening occurred before or after training, before sleep, during study-related periods, or during periods of increased academic or athletic demand.

The functional purpose of each listening episode was classified using predefined categories comprising focus, relaxation, motivation, emotional release, post-training recovery, pre-competition preparation, study support, commuting, and sleep preparation. These categories were intended to characterize why participants chose to listen to music rather than to prescribe a specific emotional-regulation strategy. Listening context was considered jointly with purpose and timing; for example, music used for motivation before training was distinguished from music used for relaxation after training or for sleep preparation in the evening.

The music-use log was maintained separately from the emotional-reflection dataset and did not contain free-text emotional-reflection fields. This procedural separation was intended to maintain methodological independence between music-related predictor variables and emotional outcome measures and to reduce direct construct overlap. Derived music-use variables included listening frequency, listening duration, time-of-day pattern, genre category, tempo category, rhythm intensity, listening context, regulatory purpose, use before or after training, use before sleep, and consistency of music use during periods of elevated academic or athletic demand. These behavioral dimensions subsequently contributed to the Music Regulation Pattern Index through timing consistency, purpose alignment, contextual alignment, and recovery-related use.

### 2.8 Nutrition rhythm and food-photo coding

Nutrition-related behavior was assessed through observation of usual eating and hydration routines. No dietary plan, calorie target, supplement protocol, macronutrient prescription, or nutrition counseling was provided. Participants recorded meal timing, hydration timing, pre-training food intake, post-training recovery intake, late-night eating, and meal regularity. Food-photo logs were used to characterize participants’ usual dietary patterns without requiring calorie estimation or a conventional quantitative dietary assessment. Participants photographed their usual eating occasions during the observation period, and the resulting images were evaluated using a structured observational coding framework based on predefined visible meal characteristics. The coding procedure focused on features that could be identified from the photographs rather than attempting to estimate exact energy intake, portion size, or nutrient composition.

Food-photo records were coded independently by two clinical sports nutritionists. Both raters were blinded to participants’ heart-rate variability, emotional scores, music-regulation values, and other outcome measures during coding. Each meal image was evaluated for meal timing, meal completeness, visible protein-containing foods, fruit or vegetable presence, carbohydrate source, hydration presence, dietary diversity, and ultra-processed food frequency. These predefined domains were used to characterize the observable composition and regularity of participants’ dietary behavior while minimizing unsupported inference about nutrients or portion sizes that could not be reliably determined from photographs alone.

Inter-rater reliability was assessed using a randomly selected 10% subset of the food-photo records, corresponding to 600 images. Agreement between the two raters was evaluated using Cohen’s kappa because the principal coding variables were categorical and kappa accounts for agreement occurring by chance. Inter-rater agreement was strong for the representative coding domains assessed, with *κ* = 0.85 for meal completeness, *κ* = 0.88 for the presence of protein-containing foods, and *κ* = 0.81 for ultra-processed food frequency. Coding discrepancies were reviewed jointly by the two raters and resolved through consensus before the finalized dataset was used in subsequent analyses.

To distinguish dietary rhythm from overall dietary quality, a separate Diet Quality Covariate was derived from the food-photo coding. This covariate incorporated visible dietary diversity, fruit and vegetable presence, protein-containing meals, and reverse-coded ultra-processed food frequency. Multilevel models evaluating the Nutrition Rhythm Stability Index were adjusted for this Diet Quality Covariate and for ultra-processed food frequency so that associations between nutrition-rhythm stability and recovery outcomes were less likely to reflect differences in overall observed dietary quality alone.

### 2.9 Emotional reflections, NLP scoring, and acoustic features

Emotional wellbeing and recovery indicators were derived from short free-text or voice-based reflections completed three times per week. Participants briefly described their current emotional state, recovery feeling, academic pressure, training condition, sleep experience, and focus level. The entries were not used for clinical diagnosis and did not include prompts about music use or nutrition behavior. Voice entries were transcribed, anonymized, and then separated from raw audio after feature extraction. All textual entries and voice transcripts were processed using Linguistic Inquiry and Word Count 2022 (LIWC-22). The Emotional Balance Score was operationalized using the LIWC-22 Emotional Tone output, where scores greater than 50 indicate a more positive emotional tone and scores below 50 indicate a more negative emotional tone. The Recovery Confidence Score was derived from LIWC-22 cognitive-process and drive-related dimensions, including insight, causation, achievement, and reward-related language.

A randomly selected 20% of entries were independently scored by two human raters using a 5-point rubric for emotional balance and recovery confidence. Inter-rater agreement was high, with ICC = 0.88 for emotional balance and ICC = 0.85 for recovery confidence. Correlations between automated and human-coded scores were *r* = 0.73 and *r* = 0.70, respectively. Acoustic features were extracted using openSMILE v3.0 with the eGeMAPS feature set, including speech rate, pause frequency, mean intensity, intensity variability, and vocal-energy variability. To reduce environmental bias, participants were instructed to record in a quiet environment when possible. Recordings with a signal-to-noise ratio below 20 dB or strong clipping were excluded. Device model and recording context were recorded and included in sensitivity checks. Acoustic variables were standardized within participant and device type before analysis. Residual variability due to smartphone microphone differences and uncontrolled recording environments was retained as a limitation.

### 2.10 Index construction and mathematical formulation

To analyze the multi-stream digital phenotyping data, three distinct analytical indices were constructed using standardized within-person z-scores to minimize baseline physiological and behavioral variance. Equal weighting was selected *a priori* to avoid overfitting and to improve interpretability. The following equations were added to improve transparency and reproducibility.

#### 2.10.1 Session rating of perceived exertion (sRPE) training load

This Equation (1) quantified internal training load by multiplying training duration by the athlete’s perceived exertion score on the modified Borg CR-10 scale.

$$sRPE\;Load=Training\;Duration\left(\min\right)\times RPE_{CR10}$$ (1)

#### 2.10.2 Music Regulation Pattern Index (MRPI)

This index integrated timing consistency, regulation purpose, contextual alignment, and recovery-related use see Equation (2).

$$MRPI_{raw}=\frac{\begin{array}{l} z\left(\mathrm{Timing}Consistency\right)+z\left(Purpose\;Alignment\right)\\ +z\left(Contextual\;Alignment\right)+z\left(\mathrm{Recovery}Use\right) \end{array}}{4}$$ (2)

#### 2.10.3 Nutrition Rhythm Stability Index (NRSI)

This index assessed meal timing regularity, hydration consistency, pre-training intake pattern, post-training recovery intake pattern, dietary diversity, and late-night eating irregularity Equation (3).

$$NRSI_{raw}=\frac{\begin{array}{l} \;\;\;\;\;\;\;\;\;z\left(\mathrm{Meal}\;\mathrm{Timing}\right)+z\left(Hydration\right)\\ +z\left(\mathrm{Pre}-Training\right)+z\left(\mathrm{Post}-Training\right)\\ +z\left(\mathrm{Diet}Diversity\right)-z\left(\mathrm{Late}-Night\;Eating\right) \end{array}}{6}$$ (3)

#### 2.10.4 Psychophysiological Recovery Balance Index (PRBI)

This index integrated autonomic recovery, physiological strain, recovery opportunity, sleep regularity, and activity-load balance see Equation (4).

$$PRBI_{raw}=\;\;\frac{\begin{array}{l} \;\;\;\;\;\;\;z\left(\mathrm{HRV}\mathrm{RMSSD}\right)-z\left(RHRDeviation\right)\\ \;\;\;+z\left(\mathrm{Sleep}Duration\right)+z\left(\mathrm{Sleep}\;\mathrm{Regularity}\right)\\ \;\;\;\;\;\;\;\;+z\left(\mathrm{Activity}-Load\;Balance\right) \end{array}}{5}$$ (4)

#### 2.10.5 Rescaling of raw index scores

To facilitate clinical and practical interpretation, each raw averaged index was linearly rescaled to a 0–100 scale using the minimum and maximum observed raw values in the sample Equation (5).

$$Index_{scaled}=\left(\frac{Index_{raw}-\min_{raw}}{\max_{raw}-\min_{raw}}\right)\times 100$$ (5)

Abbreviations used in the equations are as follows: TC, timing consistency; PA, purpose alignment; CA, contextual alignment; RU, recovery use; MT, meal timing; HY, hydration; PreT, pre-training intake; PostT, post-training intake; DD, dietary diversity; LNE, late-night eating; HRV, heart-rate variability; RMSSD, root mean square of successive differences; RHR, resting heart rate; SD, sleep duration; SR, sleep regularity; and ALB, activity-load balance. Higher scaled values indicated more favorable music regulation, nutrition rhythm stability, or psychophysiological recovery balance. Internal consistency was acceptable for MRPI (Cronbach’s alpha = 0.81), NRSI (Cronbach’s alpha = 0.78), and PRBI (Cronbach’s alpha = 0.84). Sensitivity analysis excluding the emotional-release music component produced similar associations with emotional balance, reducing the risk of construct overlap.

### 2.11 Data processing, missing data, and sensitivity analysis

All data streams were merged at the participant-day level. Quality checks were performed for wearable wear time, missing sleep episodes, extreme physiological values, duplicate music entries, unclear food photographs, missing time stamps, transcription errors, and invalid acoustic recordings. Missingness was summarized for each data stream. Complete-case models were compared with multiple imputation models using chained equations with 20 imputed datasets and predictive mean matching for continuous variables. Imputation models included age, sex, sport type, training load, academic pressure, sleep duration, HRV, RHR, diet quality, MRPI, NRSI, PRBI, and emotional markers. Multiple imputation is appropriate when the imputation model includes variables associated with missingness and outcomes.

### 2.12 Statistical analysis

Descriptive statistics were reported as mean ± standard deviation, median and interquartile range, or *n* (%), as appropriate. Because repeated daily observations were nested within participants, multilevel linear models with participant-specific random intercepts were used. Within-person centered variables were used to examine whether a participant’s deviation from their own usual music or nutrition rhythm was associated with same-day and next-day recovery and emotional markers. Models were adjusted for age, sex, sport type, session-RPE training load, sleep duration, academic pressure, competition exposure, Diet Quality Covariate, and ultra-processed food frequency when appropriate. Latent profile analysis was used to identify distinct lifestyle-regulation profiles from MRPI, NRSI, sleep regularity, session-RPE load, and PRBI. Model selection considered the Bayesian information criterion, entropy, profile size, and interpretability. The previously mentioned exploratory machine-learning analysis was removed from the revised manuscript to maintain focus on the primary prespecified observational analyses.

### 2.13 Clinical trial registration statement

This study was designed as a non-interventional observational digital phenotyping study. Participants were not prospectively assigned to music sessions, nutrition strategies, therapy, treatment, supplement use, training modification, or any health-related behavioral program. The study only observed naturally occurring music-use behavior, self-selected nutrition routines, wearable-derived recovery indicators, training load, and emotional reflection patterns during normal academic and athletic life. Therefore, clinical trial registration was not applicable. Ethical approval and written informed consent were obtained before data collection.

## 3 Results and discussion

A total of 136 medical student-athletes were screened. Sixteen participants were excluded because of incomplete baseline information, irregular sport participation, or inability to complete wearable-based monitoring (17). The final analytical sample included 120 participants, contributing 6,720 possible participant-days across the 8-week observation period. After data-quality screening, 6,048 valid participant-days were retained, corresponding to a completeness rate of 90.0% (18). This high retention indicates that non-interventional digital phenotyping is feasible in a demanding medical student-athlete population.

Figure 1 demonstrates the participant screening, inclusion, and data-retention process. Most enrolled medical student-athletes completed monitoring without receiving any assigned music session, nutrition plan, therapy, supplement, or behavioral intervention. The high completion rate supports the feasibility and low burden of the observational design.

**Figure 1:**
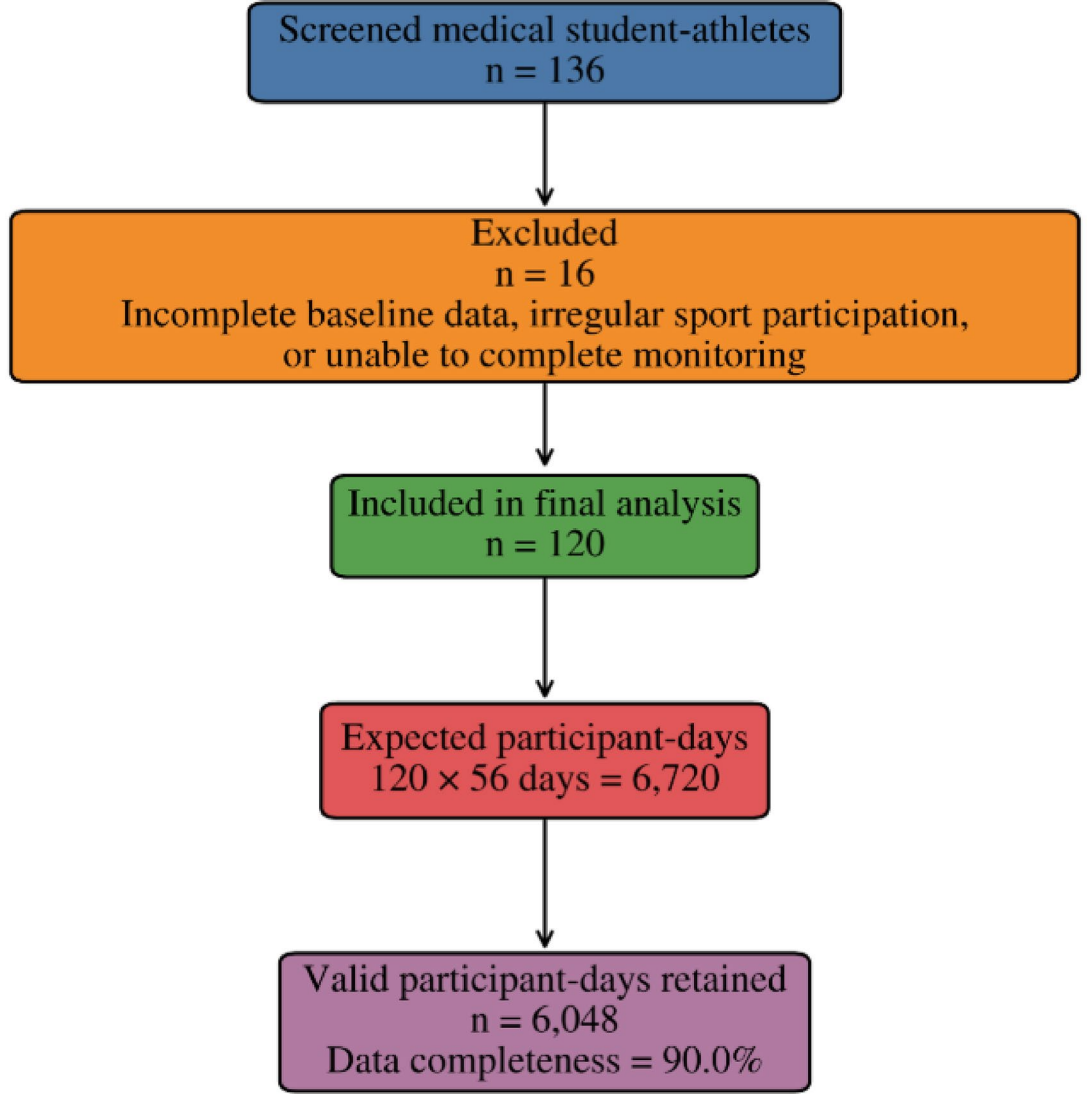
Participant flow and multimodal data completeness.

Table 1 shows that the sample included both team- and individual-sport athletes and captured participants experiencing clinical rotations and examination periods. This diversity improves ecological relevance because training rhythm, academic pressure, recovery demands, and emotional regulation strategies may differ across sport and educational contexts (19). The study integrated wearable-derived physiology, sleep–wake rhythm, training load, academic context, natural music-use records, meal timing, hydration patterns, food-photo logs, and emotional reflections. These data streams were combined into the MRPI, NRSI, and PRBI.

**Table 1:** Baseline characteristics of medical student-athletes included in the analysis.

Variable	Total sample, *n* = 120
Age, years	21.4 ± 2.1
Male participants	68 (56.7%)
Female participants	52 (43.3%)
Body mass index, kg/m^2^	22.6 ± 2.4
Weekly training frequency, sessions/week	4.6 ± 1.2
Mean training duration, min/session	82.3 ± 18.7
Team-sport athletes	71 (59.2%)
Individual-sport athletes	49 (40.8%)
Clinical rotation exposure during study	46 (38.3%)
Examination period during study	53 (44.2%)
Expected participant-days	6,720
Valid participant-days analyzed	6,048 (90.0%)

Values are presented as mean ± standard deviation or *n* (%).

Figure 2 shows the multimodal digital phenotyping framework. The framework clarifies that mental health-related and recovery markers in medical student-athletes were examined within a rhythm-based pattern involving music, nutrition, sleep, training, academic pressure, and emotional expression rather than as consequences of a single isolated behavior (20). Across the observation period, participants showed substantial variability in sleep, HRV, resting heart rate, music use, nutrition rhythm, and emotional markers. Mean sleep duration was 6.7 ± 0.9 h per night, and mean nocturnal RMSSD was 54.2 ± 13.8 ms. Participants recorded an average of 2.8 ± 1.1 music-listening episodes per day and 3.1 ± 0.7 eating occasions per day. Only 46.7% maintained stable meal timing on at least 5 days per week, and 38.3% showed irregular hydration patterns around training (Table 2).

**Figure 2:**
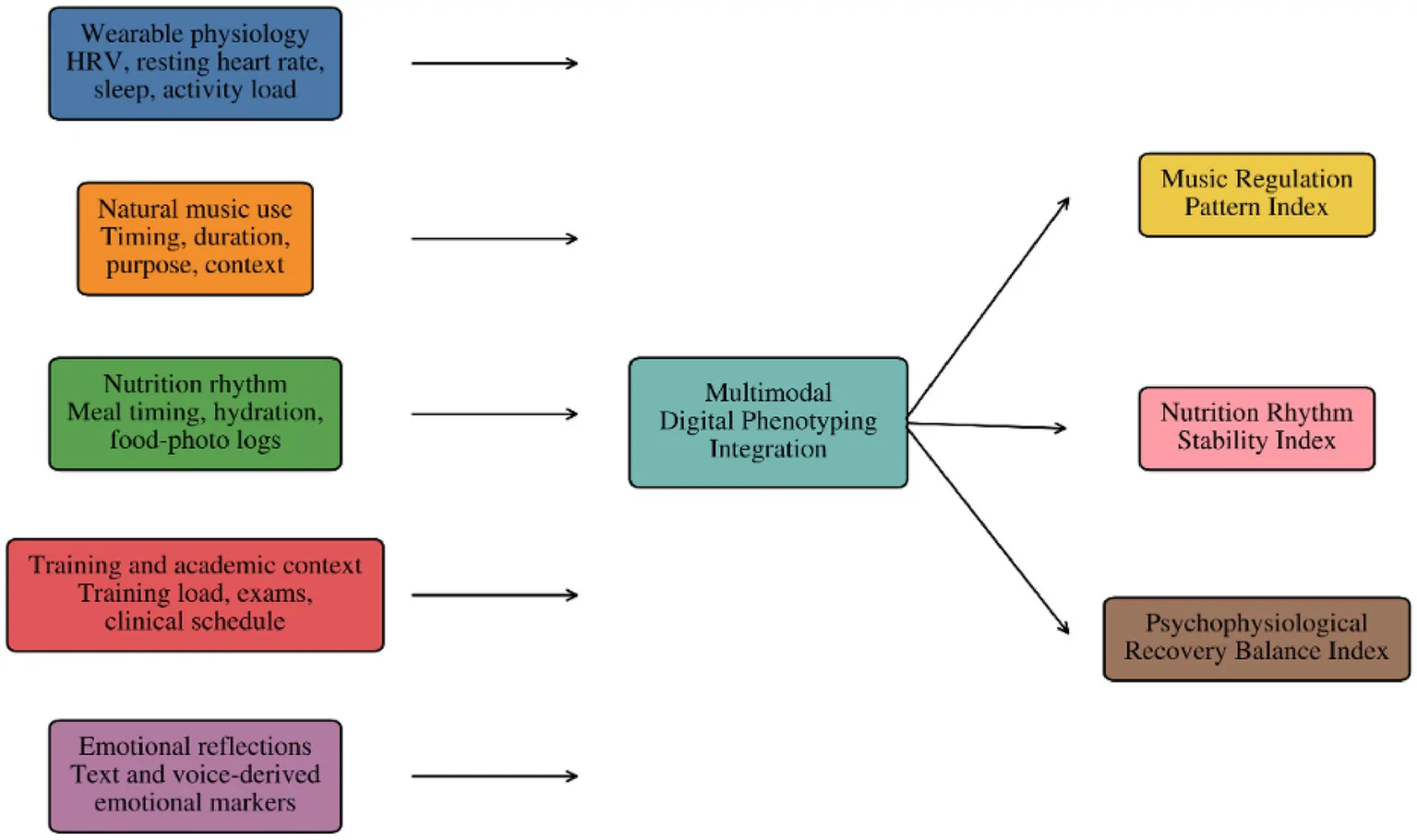
Integrated daily digital phenotyping framework.

**Table 2:** Descriptive statistics, data completeness, and reliability of digital phenotyping variables.

Variable	Mean ± SD or *n* (%)	Range/reliability
Daily sleep duration, hours	6.7 ± 0.9	4.8–8.6
Nocturnal HRV, RMSSD, ms	54.2 ± 13.8	28.5–88.9
Overnight resting heart rate, beats/min	63.7 ± 7.6	48.2–82.4
Session-RPE training load, AU	376.8 ± 124.6	120.0–760.0
Music-listening episodes/day	2.8 ± 1.1	0.5–5.6
Eating occasions/day	3.1 ± 0.7	1.8–4.9
Hydration consistency score	61.4 ± 14.2	24.0–91.0
MRPI	63.7 ± 12.9	28.4–91.6; alpha = 0.81
NRSI	61.9 ± 13.4	31.2–88.5; alpha = 0.78
PRBI	66.2 ± 11.7	35.8–92.1; alpha = 0.84
Emotional balance score	68.4 ± 12.1	38.5–91.4; ICC = 0.88
Recovery confidence score	66.7 ± 11.8	34.2–90.6; ICC = 0.85
Missing music logs	5.8%	Imputed sensitivity available
Missing nutrition logs	7.1%	Imputed sensitivity available
Missing emotional reflections	8.9%	Imputed sensitivity available

AU, arbitrary units; HRV, heart-rate variability; ICC, intraclass correlation coefficient; MRPI, Music Regulation Pattern Index; NRSI, Nutrition Rhythm Stability Index; PRBI, Psychophysiological Recovery Balance Index; RMSSD, root mean square of successive differences.

Internal consistency was acceptable for all three analytical indices. Inter-rater reliability for food-photo coding was strong for the assessed coding domains, and human-rater agreement for emotional balance and recovery confidence was high (21). Missingness was modest and did not materially change the main findings after multiple imputation. Music-use analysis showed that participants most frequently used music for relaxation, focus, motivation, post-training recovery, sleep preparation, and emotional release. Participants with higher MRPI scores used music more consistently around daily regulatory contexts such as before study, before training, after training, or before sleep (22). Those in the highest tertile of MRPI showed higher emotional balance scores than those in the lowest tertile (72.6 ± 10.4 vs. 64.1 ± 11.8).

Figure 3 indicates that relaxation, focus, and motivation contributed most strongly to the MRPI, followed by post-training recovery and sleep preparation. The revised analysis also included a sensitivity model excluding the emotional-release component to reduce concerns about overlap with emotional outcome measures; the association between MRPI and emotional balance remained significant. Nutrition rhythm analysis showed that participants with more stable meal timing, hydration consistency, and post-training intake had more favorable recovery markers (23). Participants in the highest tertile of NRSI had longer sleep duration than those in the lowest tertile (7.2 ± 0.7 vs. 6.3 ± 0.8 h) and higher nocturnal RMSSD (59.8 ± 12.6 vs. 48.7 ± 13.1 ms).

**Figure 3:**
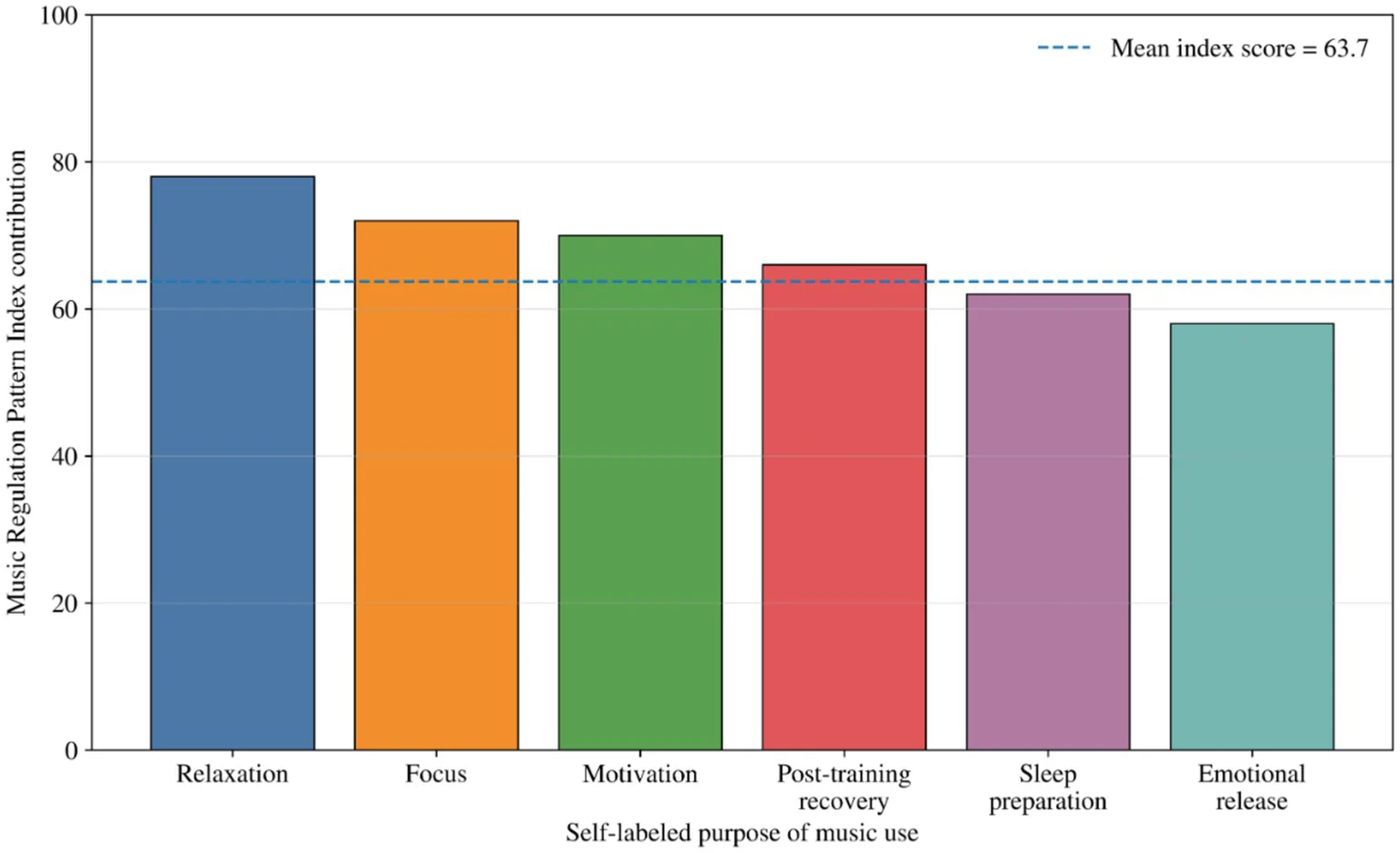
Functional categories of the Music Regulation Pattern Index.

Figure 4 shows that meal timing and dietary diversity were stronger components of NRSI, while pre-training intake and avoidance of late-night irregular eating were weaker. Importantly, the association between NRSI and recovery balance remained significant after adjustment for overall Diet Quality Covariate and ultra-processed food frequency, suggesting that rhythm stability was not only a proxy for higher dietary quality (24). Weekly wearable patterns showed that recovery balance declined during high academic-pressure and competition weeks. During high academic-pressure weeks, mean sleep duration decreased from 6.9 ± 0.8 to 6.2 ± 0.9 h, overnight resting heart rate increased by 4.3 ± 2.1 beats per minute, and nocturnal RMSSD declined by 7.6 ± 3.5 ms. These findings suggest that academic pressure and sport demand may interact to reduce recovery capacity (25).

**Figure 4:**
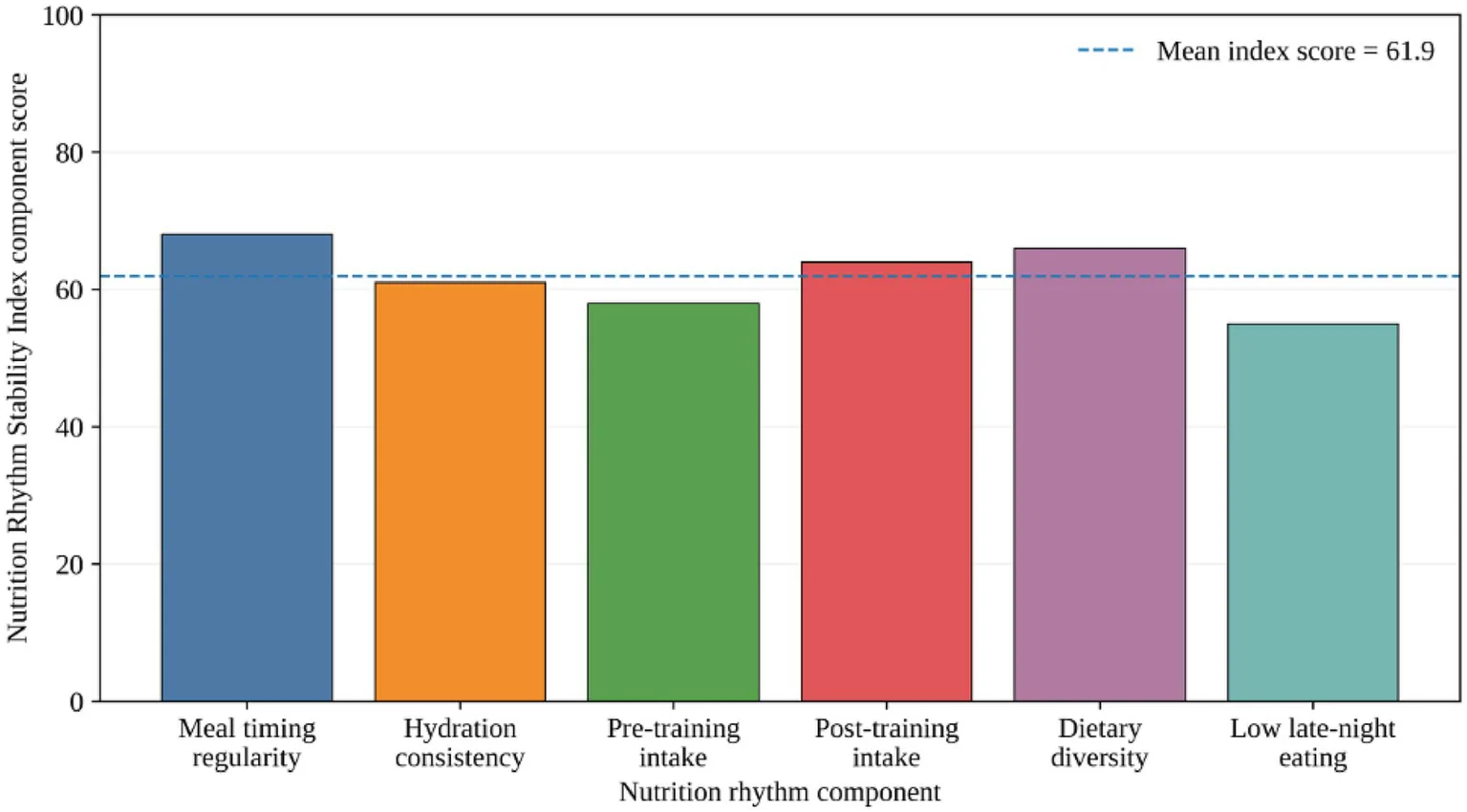
Nutrition Rhythm Stability Index and recovery-supportive eating patterns.

Figure 5 illustrates weekly changes in sleep duration, HRV, resting heart rate, and recovery balance. Weeks with higher academic or competition demand showed reduced sleep and HRV with increased resting heart rate. This pattern supports the view that recovery in medical student-athletes is both a sports-performance and academic-health issue.

**Figure 5:**
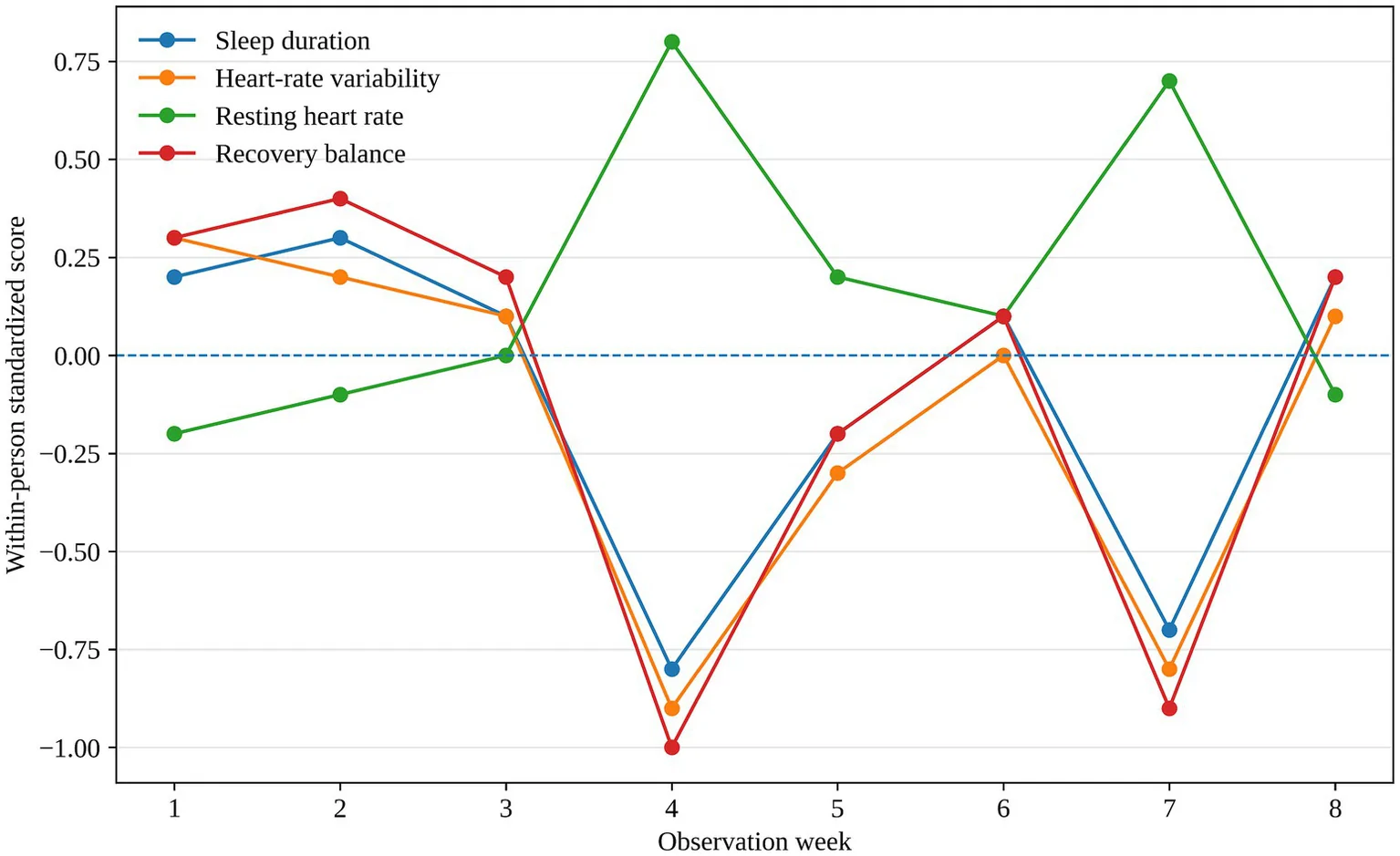
Weekly variation in sleep, heart-rate variability, resting heart rate, and recovery balance.

Correlation analysis demonstrated meaningful associations among lifestyle rhythm, wearable recovery, and emotional markers (26). MRPI was positively correlated with emotional balance, recovery confidence, and focus-related language. NRSI was positively correlated with sleep regularity, HRV stability, and PRBI. PRBI was positively associated with emotional balance and negatively associated with stress-related language (11).

Figure 6 suggests that music and nutrition may represent complementary self-regulation pathways. Music regulation was more closely associated with emotional and cognitive markers, whereas nutrition rhythm stability was more closely associated with sleep and physiological recovery. This distinction supports analyzing both domains rather than treating them as interchangeable behaviors.

**Figure 6:**
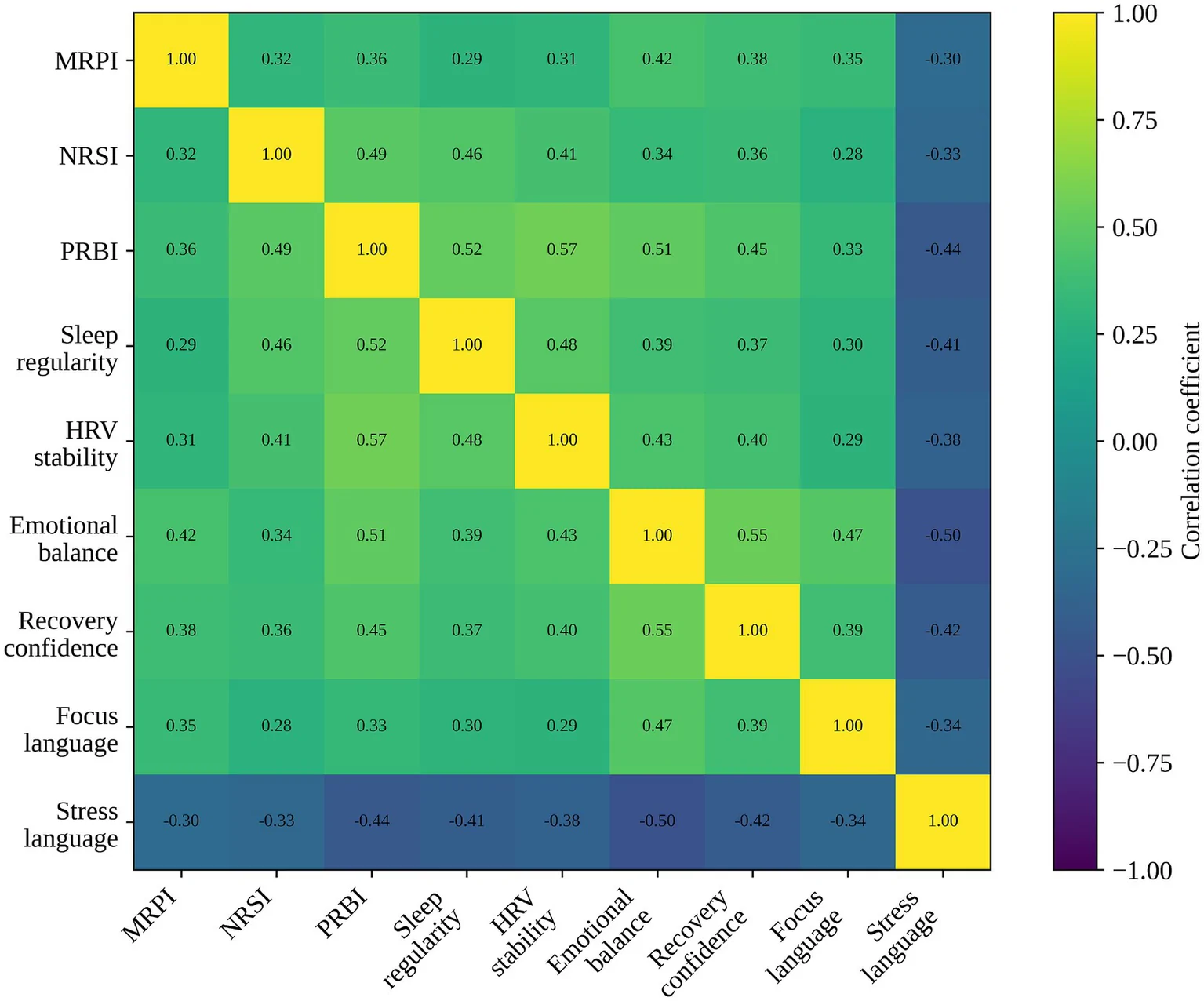
Correlation matrix among music regulation, nutrition rhythm, wearable recovery, and emotional markers.

Multilevel models accounted for repeated participant-days nested within individuals. After adjustment for age, sex, sport type, session-RPE training load, sleep duration, academic pressure, competition exposure, Diet Quality Covariate, and ultra-processed food frequency, MRPI remained associated with emotional balance and focus-related language (27). NRSI remained associated with PRBI, sleep regularity, and HRV stability (28). Complete-case and multiple-imputation models produced similar coefficients; the largest coefficient difference was 0.03, and no primary inference changed (Table 3).

**Table 3:** Multilevel model estimates and sensitivity analyses.

Predictor	Outcome	Complete-case beta	MI beta	95% CI	*p*-value
MRPI	Emotional balance	0.29	0.28	0.18 to 0.40	<0.001
MRPI	Focus-related language	0.24	0.23	0.12 to 0.35	<0.001
MRPI	Recovery confidence	0.22	0.21	0.10 to 0.34	0.001
NRSI	Recovery balance	0.34	0.33	0.22 to 0.46	<0.001
NRSI	Sleep regularity	0.31	0.30	0.19 to 0.43	<0.001
NRSI	HRV stability	0.27	0.26	0.15 to 0.39	<0.001
Evening music use	Next-day emotional balance	0.21	0.20	0.09 to 0.33	0.002
Post-training intake	Next-day recovery balance	0.26	0.25	0.14 to 0.38	<0.001
MRPI excluding emotional-release subcomponent	Emotional balance	0.26	0.25	0.15 to 0.37	<0.001
NRSI adjusted for diet quality and ultra-processed foods	Recovery balance	0.31	0.30	0.19 to 0.43	<0.001

Models were adjusted for age, sex, sport type, session-RPE training load, sleep duration, academic pressure, competition exposure, Diet Quality Covariate, and ultra-processed food frequency where appropriate (29). MI, multiple imputation; HRV, heart-rate variability.

Figure 7 visually summarizes the primary standardized coefficients. The revised results now report missing-data sensitivity analysis and demonstrate that multiple imputation did not materially alter the main findings. The independent data streams used to build MRPI and emotional outcomes reduce the risk of circularity, and the MRPI sensitivity model excluding emotional-release labeling produced consistent results.

**Figure 7:**
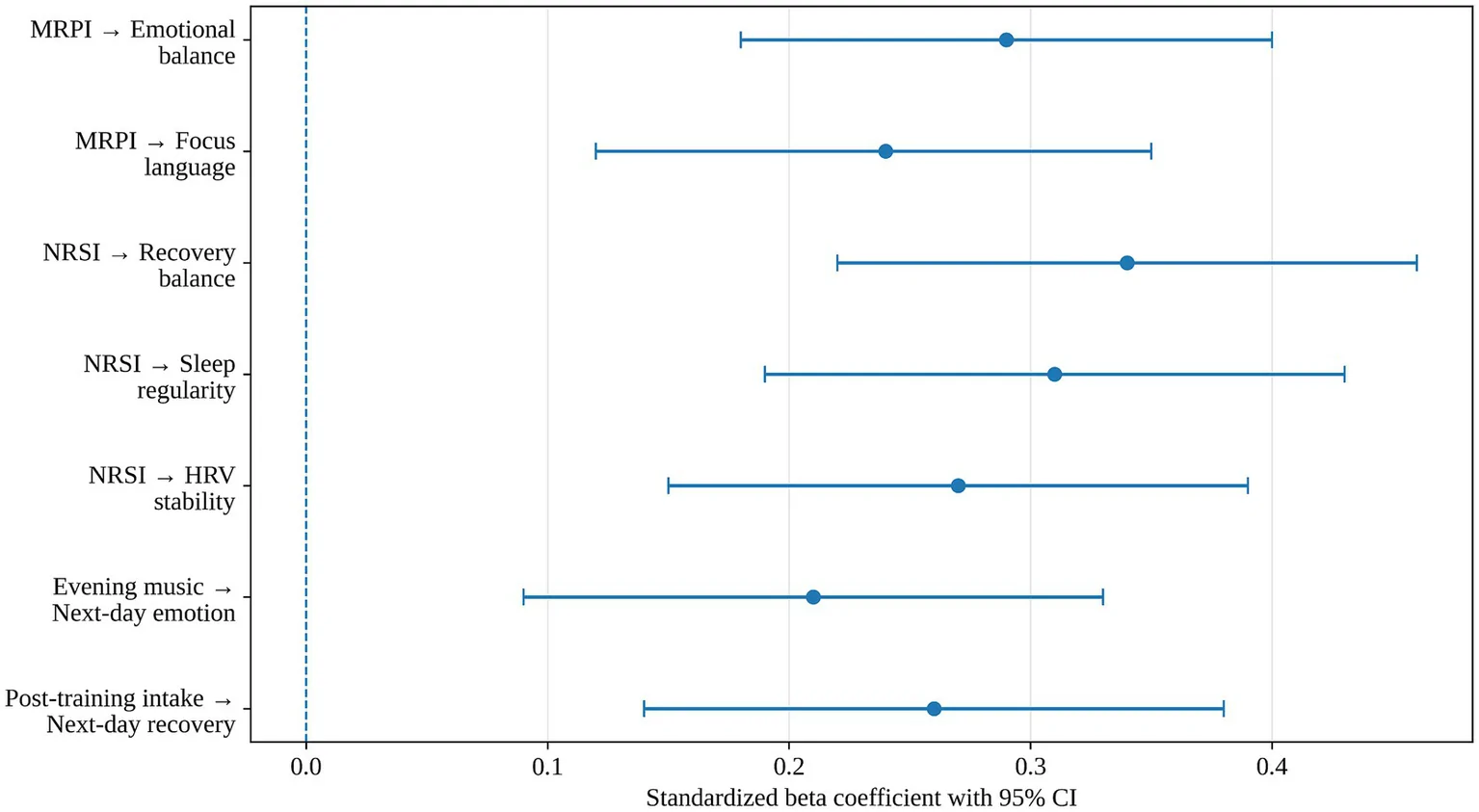
Multilevel model estimates for daily and next-day recovery and emotional outcomes.

Latent profile analysis identified four lifestyle-regulation profiles. Profile 1, stable music-nutrition regulation, included 31.7% of participants and was characterized by high MRPI, high NRSI, regular sleep, and favorable recovery balance (30). Profile 2, high training load with preserved recovery, included 24.2%. Profile 3, irregular nutrition and reduced recovery, included 25.8%. Profile 4, low regulation and high emotional strain, included 18.3% (Table 4).

**Table 4:** Latent lifestyle-regulation profiles and recovery-emotional outcomes.

Profile	n (%)	Emotional balance	Recovery confidence	Recovery balance	Stress-language score
Stable music-nutrition regulation	38 (31.7%)	76.8 ± 8.7	74.5 ± 8.9	73.9 ± 9.2	21.0 ± 6.8
High training load with preserved recovery	29 (24.2%)	71.2 ± 9.5	69.8 ± 9.7	70.1 ± 9.6	27.5 ± 7.4
Irregular nutrition and reduced recovery	31 (25.8%)	64.6 ± 10.8	62.9 ± 10.2	60.8 ± 10.6	38.8 ± 8.9
Low regulation and high emotional strain	22 (18.3%)	58.9 ± 11.4	56.4 ± 11.1	54.7 ± 11.8	49.5 ± 10.3
Overall comparison	–	*F* = 18.72	*F* = 16.84	*F* = 19.35	*F* = 21.08
*p*-value	–	<0.001	<0.001	<0.001	<0.001

Figure 8 shows that the stable music-nutrition regulation profile had the most favorable pattern across emotional balance, recovery confidence, and recovery balance, whereas the low-regulation/high-strain profile had the least favorable pattern. These profiles may help medical schools and sport departments identify patterns of risk and resilience without treating the findings as causal intervention evidence.

**Figure 8:**
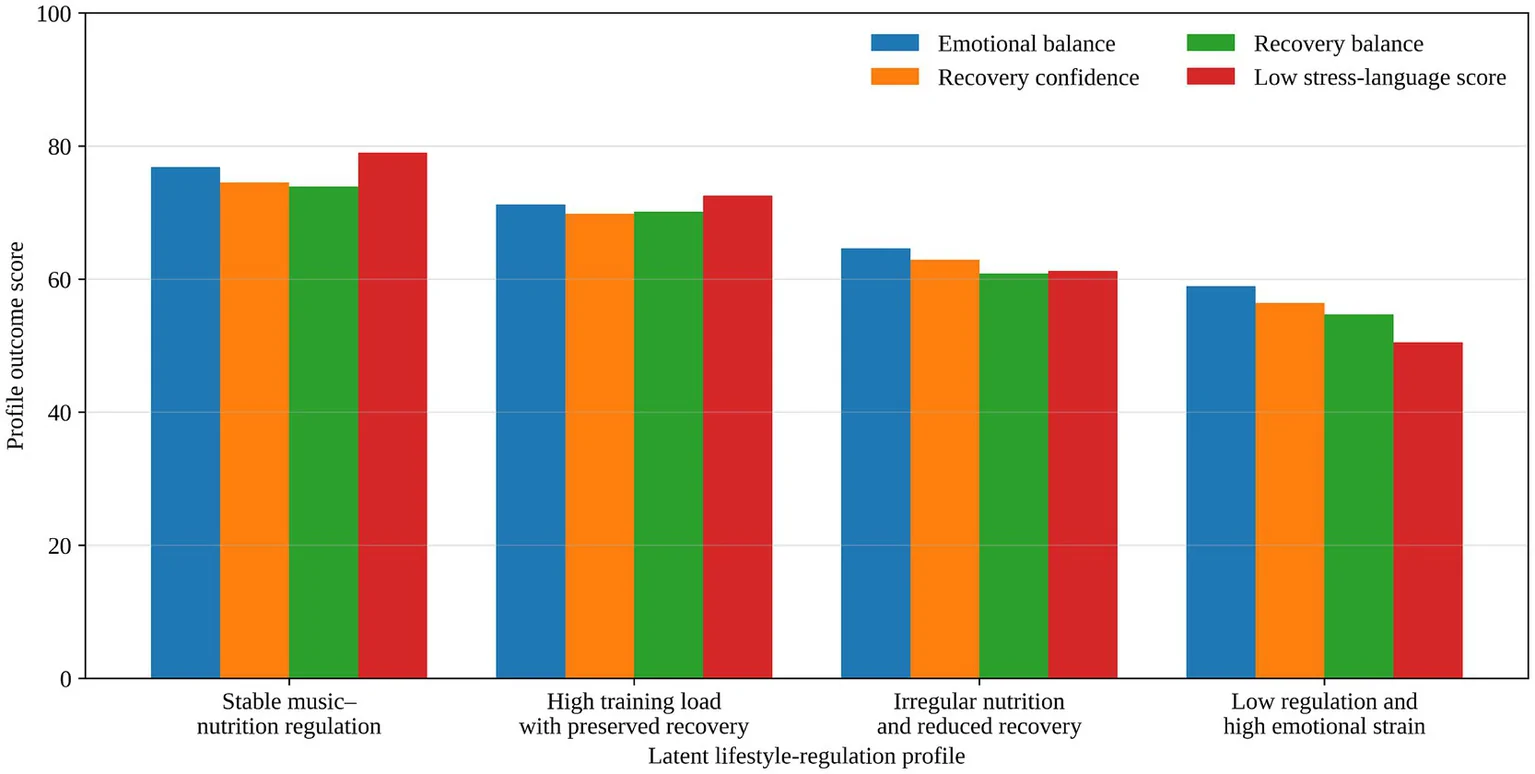
Latent lifestyle-regulation profiles and emotional recovery outcomes.

### 3.1 Expanded practical interpretation

The practical implication of these observational profiles is not that medical schools should immediately prescribe music or diet programs (31). Rather, the results suggest low-burden support targets that can be developed and later tested in registered intervention studies. For nutrition rhythm, medical schools could support regular eating by providing protected micro-breaks during clinical rotations, accessible hydration stations, portable recovery snacks after training, and scheduling guidance that helps athletes plan pre- and post-training intake around irregular clinical duties (32). For music-based regulation, schools could provide quiet recovery spaces, optional self-selected music resources, and education about using music for transition periods such as pre-study focus, post-training decompression, and sleep preparation (33). Because these strategies would become active behavioral supports if assigned prospectively, future efficacy testing should be conducted as a registered trial.

### 3.2 Limitations

Several limitations should be acknowledged. First, the observational design prevents causal inference. The study cannot conclude that music regulation or nutrition rhythm directly improves mental health. Another limitation relates to participant burden associated with prolonged multimodal monitoring. Although wearable measurements reduced the need for continuous manual reporting of physiological variables, participants were required to actively record daily music-use behaviors, meal timing, hydration patterns, food photographs, training information, and emotional reflections throughout the eight-week observation period. This intensive tracking process may contribute to response fatigue, delayed data entry, incomplete reporting, or retrospective logging when immediate recording was not possible. Consequently, active behavioral datasets may contain recall bias or reduced temporal accuracy despite the implemented data-quality checks and missing-data sensitivity analyses. Future studies should consider automated passive sensing approaches or reduced-burden reporting strategies to improve ecological validity and participant adherence.

Second, although the wearable device provides practical real-world physiological data, consumer devices cannot fully replace electrocardiography or polysomnography. Third, although food-photo coding demonstrated acceptable inter-rater reliability in a randomly selected 10% subset of images (600 photographs), the remaining majority of dietary images were coded by a single reviewer after the coding framework was established. Therefore, individual interpretation bias and potential coding drift cannot be completely excluded for approximately 90% of the food-photo dataset. In addition, image-based coding may not accurately capture portion size, hidden ingredients, or micronutrient intake. Fourth, emotional reflection scores were derived from text and voice features rather than clinical diagnostic interviews. Fifth, acoustic features are sensitive to recording environment and smartphone microphone differences; although low-quality recordings were excluded and device type was considered, recording conditions were not fully controlled. Another limitation concerns the characterization of music exposure. Because copyrighted audio files were neither collected nor stored, the study relied on participant-reported music characteristics, including genre category, tempo category, perceived rhythm intensity, and listening purpose. Therefore, direct acoustic analysis of individual tracks could not be conducted to objectively verify musical features or quantify standardized exposure characteristics across participants. Future studies incorporating secure audio-feature extraction methods or non-identifiable acoustic descriptors may allow more objective validation of musical parameters while maintaining participant privacy. Finally, the sample was limited to medical student-athletes, so findings may not generalize to professional athletes, non-medical students, or clinical populations.

## 4 Conclusion

This study presents a non-interventional multimodal digital phenotyping framework for examining mental health-related and recovery markers among medical student-athletes. By specifying the wearable device, signal windows, software pipeline, mathematical index formulas, food-photo reliability, emotional-scoring procedure, missing-data sensitivity analysis, and confounding control, the revised study improves reproducibility and addresses key methodological concerns. The findings suggest that consistent music-based emotional regulation is more closely associated with emotional balance and focus-related markers, whereas stable nutrition rhythm is more strongly associated with sleep regularity, nocturnal HRV stability, and recovery balance. Medical student-athletes with stable music-nutrition regulation profiles showed the most favorable recovery-emotional outcomes, while those with low regulation and irregular routines showed greater emotional strain. Because the study is observational, these results should be interpreted as associations rather than treatment effects. The framework may help universities and sport departments identify low-burden wellness-support targets, but future registered intervention trials are needed to test whether structured music or nutrition-rhythm strategies can actively improve mental health and recovery outcomes.

## Data Availability

The raw data supporting the conclusions of this article will be made available by the authors, without undue reservation.
